# Multifocal intra-parenchymal and sub-pectoral malignant phyllodes tumor in young female, rare and unusual presentation: A case report

**DOI:** 10.1016/j.ijscr.2020.05.014

**Published:** 2020-05-22

**Authors:** Mohammad Naser Athamnah, Omar M. Abuelaish, Nimah A. Rabai

**Affiliations:** a Jordan Board of General Surgery, Jordan Medical Council, Jordan; b Breast Surgery Fellowship, San Gerardo Hospital, Milano, Italy; c Breast Surgeon, Jordan Ministry of Health, Jordan; d Fellow of the American College of Surgeons, United States; e Breast Surgeon, Jordan Royal Medical Services, Jordan; f General Surgery Resident, Jordan Ministry of Health, Jordan

**Keywords:** Malignant phyllodes tumor, Sub-pectoral phyllodes, Multifocal phyllodes, Immediate reconstruction

## Abstract

**Introduction:**

Phyllodes tumors are rare fibroepithelial breast tumors, accounting for less than 1% of all breast tumors. Most Phyllodes tumors are benign. However, about 10% are malignant. The Mean age of presentation for the Malignant Phyllodes tumors is 40 years. This case report describes an unusual presentation of Phyllodes tumor in a young female with an unusual site and presentation of the tumor.

**Presentation of case:**

A 23-year-old female presented with multiple left breast rapidly growing masses. Imaging showed two intra-parenchymal left breast masses and one mass lying posterior to the left pectoralis major muscle, anterior to the chest wall. Diagnosed as phyllodes tumor. Nipple areola sparing mastectomy was performed with excision of the tumor and immediate reconstruction using a silicone implant, followed by radiotherapy.

**Conclusion:**

Phyllodes tumor usually presents in patients between 35–55 years old as a single breast mass. Yet, in rare cases, multifocal disease can happen, and disease can present in younger ages. The sub-pectoral presentation of phyllodes tumor is not mentioned in the literature before this case.

## Highlights

• To the best of our knowledge, the current report is the first to mention Sub-pectoral breast primary phyllodes tumor.
• Phyllodes tumors usually present in 35–55 years old female patients but should be suspected in any fast-growing breast mass, even in young age groups.
• In this case the patient preferred adjuvant radiotherapy to the nipple-areola complex and surrounding skin instead of surgical excision although results are oncologically inferior.
• Early diagnosis and initiation of appropriate treatment of Phyllodes tumors may lead to prevent metastasis and improve outcomes.

## 1 Introduction

Phyllodes tumors are rare fibroepithelial tumors of the breast, accounting for less than one percent of all breast tumors [[1], [2], [3]]. Phyllodes tumors are divided into three categories: benign, borderline and malignant phyllodes with the majority of cases defined as benign [4]. It is unusual for a malignant phyllodes tumor to present in young patients [2]; for, the mean age of presentation of malignant Phyllodes tumor is 40 years [5]. Even though both breasts have the same propensity of phyllodes tumor occurrence, phyllodes tumors generally manifest as a rapidly growing single breast mass [6,7] that is commonly located in the upper outer quadrant of the breast [8]. Multifocal or bilateral presentations of phyllodes tumors are uncommon and most often are asynchronous and cancerous [1].

As the surgical excision is the treatment of Phyllodes tumor with a safety margin of 1 cm mastectomy and conservative breast surgery are both valid options [9]. In addition, adjuvant radiotherapy is advised in cases of high malignancy and incomplete excisions. Younger age patients with sizable masses might also benefit from radiotherapy. Chemotherapy, however, has no significant effect on the treatment of Phyllodes tumors [9]. Phyllodes Tumor can reach huge dimensions. Local recurrence rates are related to tumor size. However, tumor size does not seem important in determining metastatic potential. Unlike epithelial breast cancers, Phyllodes tumors do not usually metastasis to axillary lymph nodes, and therefore, axillary dissection is not warranted in Phyllodes tumor unless there is proven metastasis [10]. An unusual case of phyllodes tumors is discussed here.

## 2 Case presentation

**Fig. 1:**
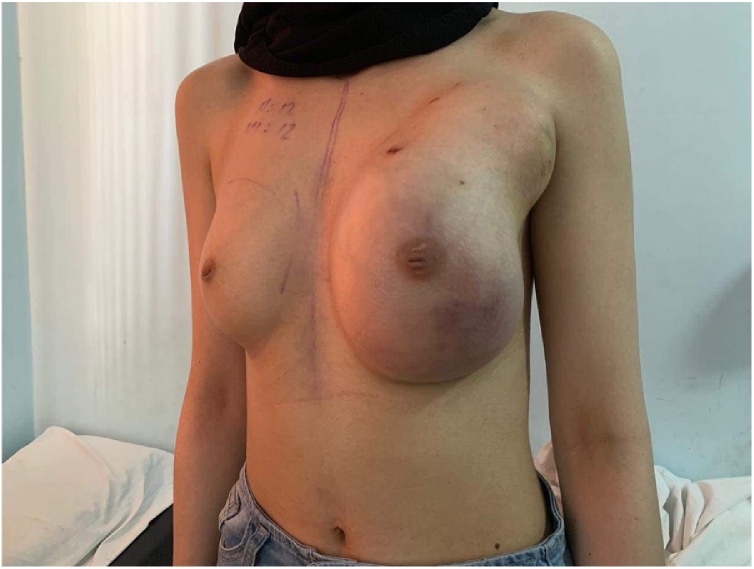
Picture showing pre-operative left Breast tumor.

**Fig. A1:**
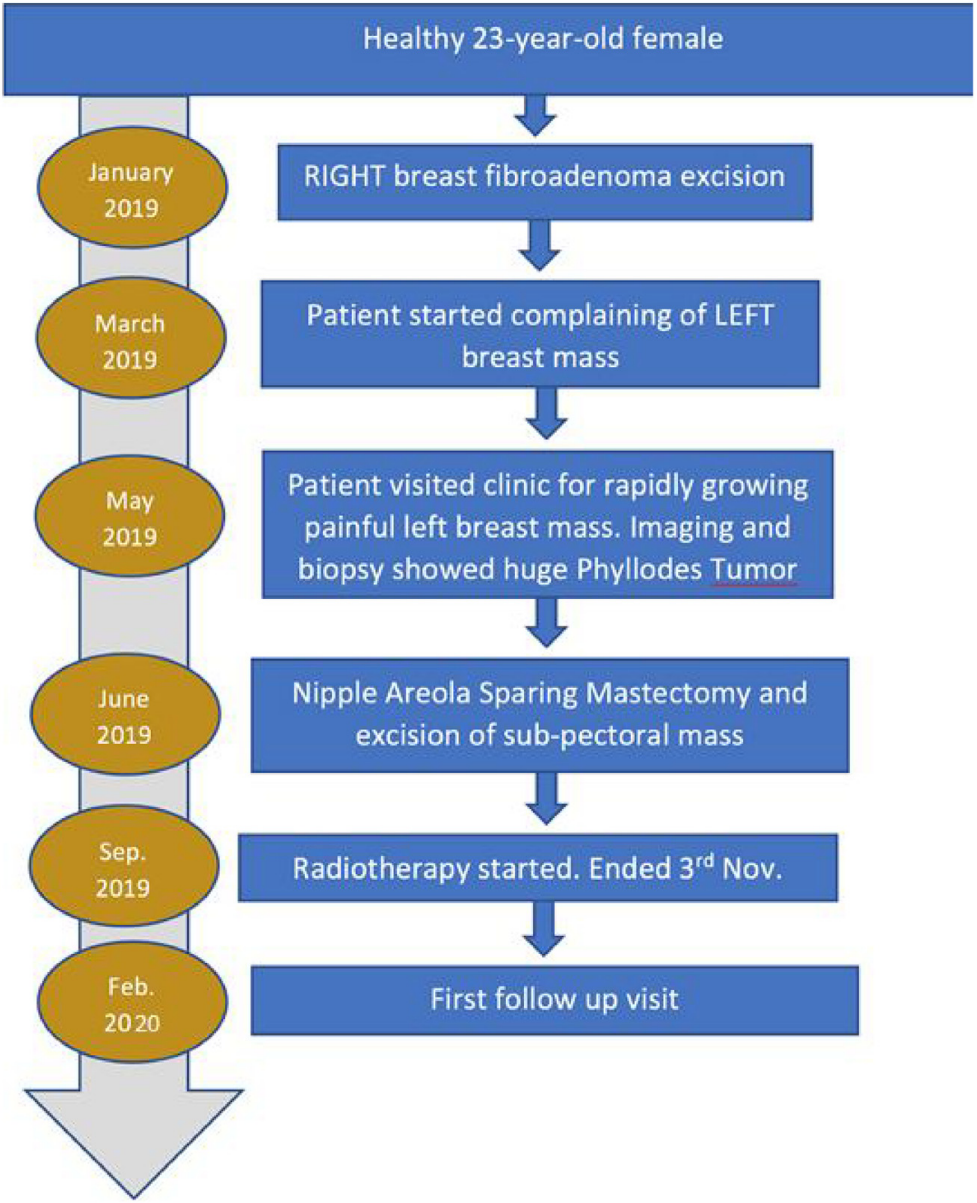
The time for diagnosis and treatment.

A 23-year-old woman with no known risk factors for breast and/or ovary cancer who had a fibroadenoma excision under local anesthesia in Jan 2019 was presented to our hospital because of left breast mass. The mass was first noticed in March 2019 and was increasing in size dramatically, growing from the breast towards the armpit over the next two months (see Fig. A1 in the Appendix A section for timeline). The fast-growing mass was deep, hard and minimally mobile. It was associated with skin redness and engorgement of the breast. There was no nipple discharge. There were no other masses noticed in the right breast or any other site (Fig. 1). There were no systemic symptoms like fever, general weakness or change in body weight. By May 2019 the mass started to feel painful and the patient was unable to sleep without taking NSAIDs. Hence, the patient visited the surgery clinic. Upon physical exam, the young lady looked well, with stable vital signs. Her right breast showed longitudinal scar lateral to areola related to the previous excision of fibroadenoma. There were no palpable masses on the right side. The left breast was markedly enlarged, especially in the outer quadrant, with a small patch of skin discoloration (necrosis) measuring around 1 cm in the upper inner quadrant above the nipple. Many dilated superficial veins were seen, with no nipple discharge. On palpation of left breast, two masses were felt: one parenchymal measuring almost 10 × 8 cm, and the other deep between breast tissue and axilla (muscular) with ill-defined inner borders. Both masses were hard and immobile, and they were fixed to underlying structures. No palpable axillary lymph nodes were found.

The rest of her physical exam results were within normal limits. Her blood and laboratory results of complete blood count, kidney function tests, and liver function tests were all within normal limits.

**Fig. 2:**
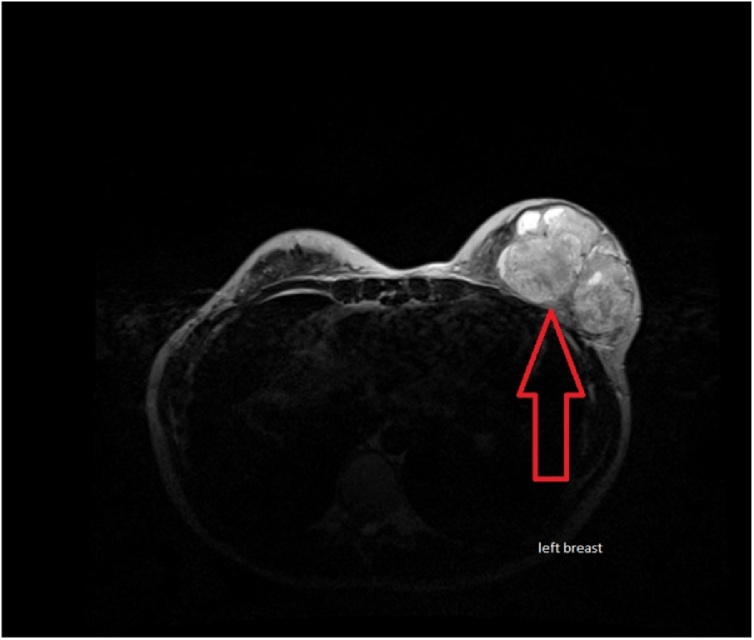
Breast MRI. Arrow pointing towards bilobed intra-parenchymal Phyllodes tumor.

**Fig. 3:**
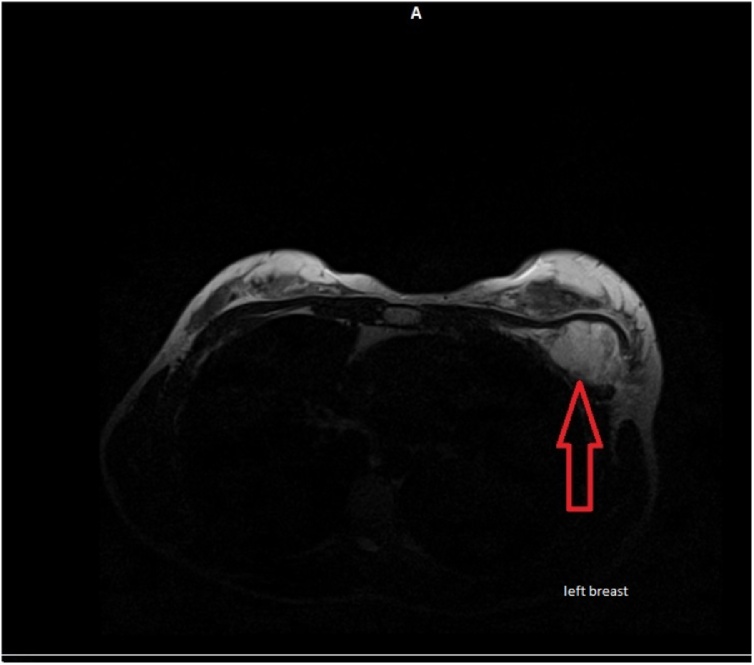
Breast MRI. Arrow pointing towards sub-pectoral Phyllodes tumor.

**Fig. 4:**
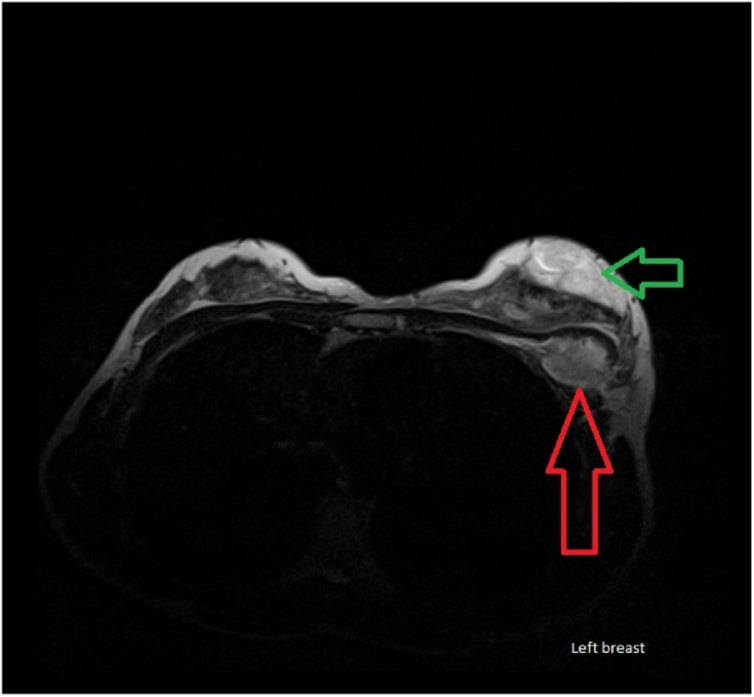
Breast MRI. Multifocal intra-parenchymal and sub-pectoral phyllodes tumor. Green arrow points towards intra-parenchymal Phyllodes tumor, red arrow points towards sub-pectoral Phyllodes tumor.

Breast ultrasound and breast MRI were performed on the 11th of May 2019 and showed normal right breast and multiple left breast lesions. Lesions' appearance was suggestive of bilobed/multifocal intra-parenchymal phyllodes tumor (Fig. 2). A well-circumscribed mass located between the anterior chest wall and pectoralis major muscle was seen (Fig. 3). The impression of a multifocal intra-parenchymal and sub-pectoral phyllodes tumor was concluded by MRI imaging (Fig. 4). Histopathological study of left breast mass Tru-Cut biopsy performed on the 27th of May 2019 showed phyllodes tumor of borderline category: it featured an adequate sample of tissue with fibro-adenomatoid changes showing prominent stromal cellularity, occasional mitoses, and no apparent stromal atypia. A pan CT scan revealed no radiological evidence of distant metastatic disease.

**Fig. 5:**
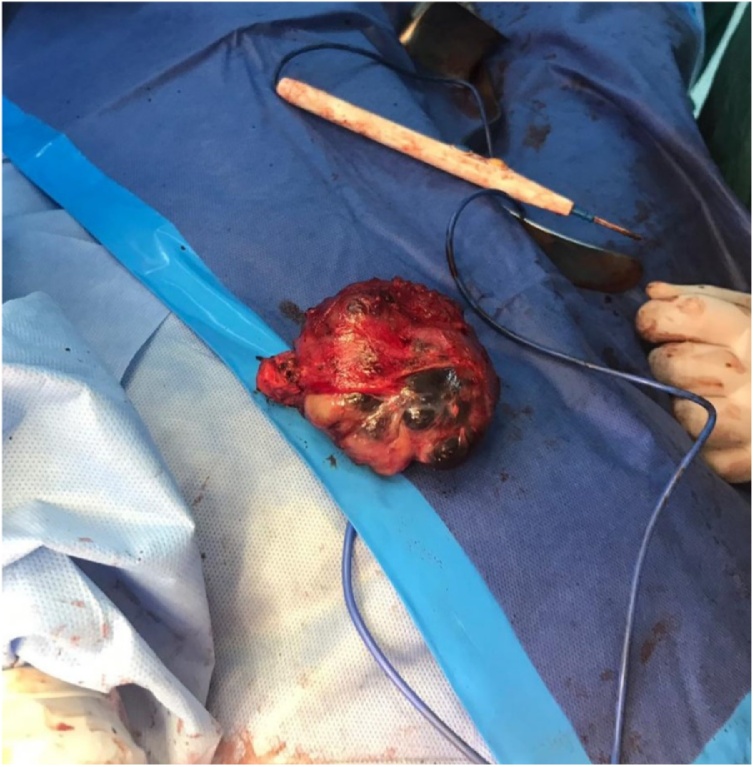
Intraoperative picture of the retro-pectoral mass after complete excision.

The following surgery was performed on the 3rd of June 2019: left breast nipple-sparing mastectomy with the excision of a retro-pectoral mass (Fig. 5) and immediate breast reconstruction using a submuscular Silicone Implant (305 cc Mentor™ anatomical implant) & Ti-Loop™ Mesh.

**Fig. 6:**
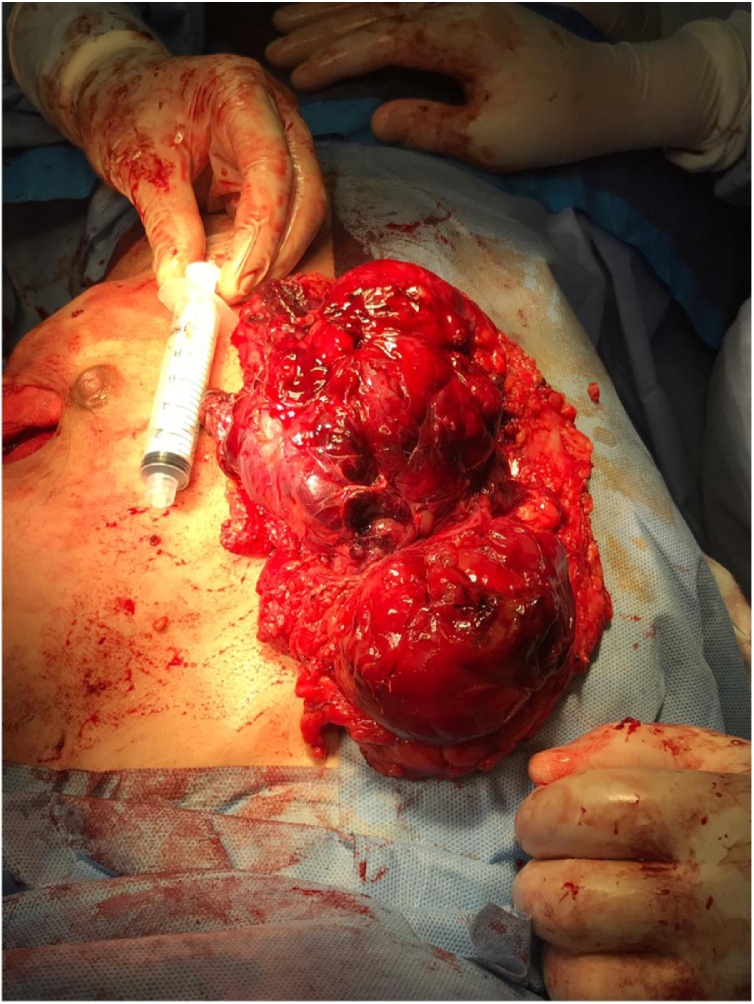
Mastectomy specimen with two intraparenchymal masses.

Mastectomy specimen showed a 16 × 14 × 10 cm mass, comprised of two tumors, (Fig. 6). First one is 8 × 7 × 6 cm, and the second one is 12.5 × 8 × 7.5 cm. Microscopic examination showed hypercellular stroma with atypia, the mitotic rate is high and about more than >10 mitotic figure per 10 high per field with atypical mitosis, with marked stromal overgrowth with focal osteoid matrix identified. The densely packed anaplastic stromal cells are infiltrating into adjacent borders, representing a malignant phyllodes tumor, that is incompletely excised. The 1st tumor is reaching the anterior and lateral margins (subareolar/cutaneous margin) 0.6 cm superior margin, posterior and medial margins are free (more than 1 cm away). The second tumor is also reaching the anterior margin (subareolar/cutaneous margin), Posterior margin (chest wall) is 0.1 cm away, lateral and medial margins are free (more than 1 cm away). Both, Heterologous elements (osteoid) and stromal overgrowth are identified. Histopathology for retro-pectoral mass showed an 8 × 7 × 6 cm malignant phyllodes tumor that is excised with 0.1 cm superior margin and 0.2 cm for both lateral and medial margins.

**Fig. 7:**
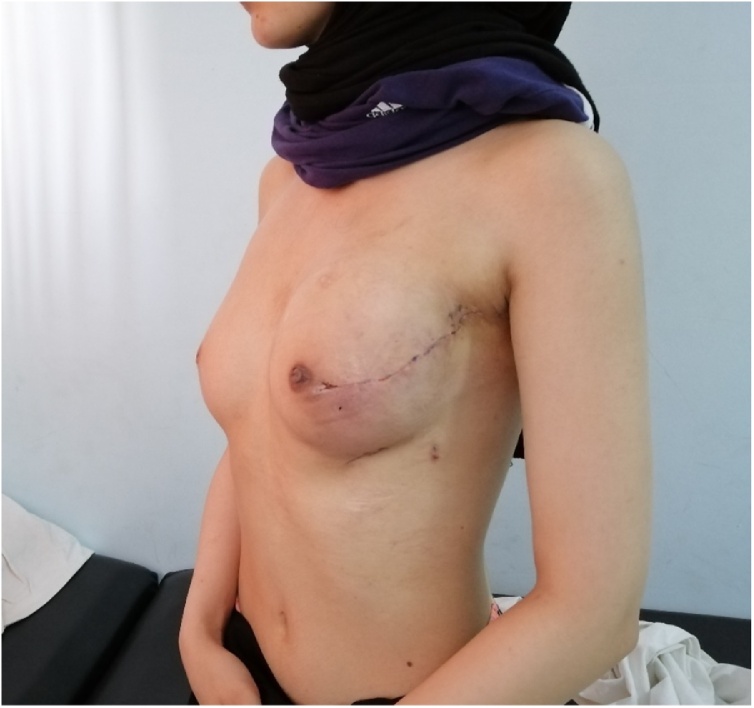
Post-operative Left Breast picture.

One-month post-operation and after full patient recovery (Fig. 7), the patient was sent to a radiotherapy unit at another medical center for assessment and consultation. The patient was planned for adjuvant radiotherapy to the chest wall: 2.67 Gy over 33 sessions starting on the 16th of September and finishing by the 3rd of November 2019.

The patient is now scheduled for 6-month-interval follow-up for local recurrence and distant metastatic workup.

## 3 Discussion

The current report presented a case of malignant Phyllodes tumor that is exceptional in several aspects: age, multifocality and the extraordinary site of the tumor. Adding more complexity to the situation, patient refused the radical surgery of removing nipple areola complex.

Malignant phyllodes tumors are rare and peak usually in the fourth decade of life. However, it can occur at a younger age. Presentation of malignant Phyllodes tumor is uncommon at a young age. Only a few cases of malignant Phyllodes tumors in younger age groups were reported. About twenty cases of children of Phyllodes tumors have been previously reported [[11], [12], [13], [14], [15], [16], [17], [18], [19]]. Of those cases, only three were malignant [13,18,19]. For young women between 20–29 years old, a previous study by Abusalem and AlMasri [20] indicated that four out of 26 patients with Phyllodes tumors were in their twenties. Of those four, one had a malignant tumor. In addition, a recent study by Nguyen et al. [21] reported a high-grade malignant Phyllodes tumor in a 26-year-old female. Early diagnosis and initiation of appropriate treatment of Phyllodes tumors may lead to prevent metastasis and improve outcomes.

Phyllodes tumor is usually described by patients as a single fast-growing breast mass. Phyllodes tumor generally represents a single mass with well-defined borders [22]. In the case reported here, the presence of three masses increasing in size simultaneously in a short period indicates a unique presentation regarding multifocality.

To the best of our knowledge, cases of extra-parenchymal primary Phyllodes tumor of the breast have not been studied before. In this case report, the 23-year-old female had a unique presentation of a fast-growing Phyllodes tumor between the pectoralis major muscle and anterior chest wall, next to this fast-growing mass, two additional masses with almost the same size were found at the left breast intra-parenchymal area. All three masses were diagnosed later as Malignant Phyllodes tumors. Malignant Phyllodes tumors have the potential to distantly metastasize [23]. The presence of local extramammary Phyllodes tumor in the current case is not easy to understand or explain as it is the first time for a such presentation to be reported.

Complete surgical excision of Phyllodes tumor is the treatment of choice. Involved margins increase the risk for local recurrence. Conservative or radical surgeries are all valid options [24]. Phyllodes tumors are not more aggressive in younger women compared to older patients [25]. The patient in the current report refused any surgical intervention that would leave her with a deformity, including nipple-areola complex removal. She insisted on preserving her natural nipple and areola despite the presence of the tumor. Her young age, the stigma of nipple-areola complex removal and the psychological impact were given as reasons behind declining any surgery involving nipple-areola complex removal. The patient defended her decision to avoid redo surgery for complete excision depending on the available evidence that post-operation radiotherapy decreases local and systemic recurrence of the disease [9]. She also argued that even in cases of complete excision, local or distant recurrences are around 20 % for malignant Phyllodes [26]., The patient also expressed concern of misshapenness and lower self-esteem as an additional reason for refusing nipple areola complex removal. After completion of radiotherapy treatment, the patient is now scheduled for her 6-month-interval routine follow-up. The follow up will involve a general exam and detailed breast exam that will include chest CT scan as recommended by Tremblay-LeMay et al. [27].

## 4 Conclusion

Phyllodes tumors usually present in 35–55 years old female patients but should be suspected in any fast-growing breast mass, even in young age groups. Phyllodes Tumors are usually mono-focal and unilateral with multifocal cases being very rare. The current report is the first to include Sub-pectoral breast primary phyllodes tumor. Finally, in this case the patient preferred adjuvant radiotherapy to the nipple-areola complex and surrounding skin instead of surgical excision although results are oncologically inferior. Early diagnosis and initiation of appropriate treatment of Phyllodes tumors may lead to prevent metastasis and improve outcomes.

## Funding

No funds received.

## Ethical approval

No ethical approval is needed for case reports as long as we have written patient consent and patient is anonymised.

## Consent

I have written consent signed by the patient. Printed and signed in patient native language: Arabic.

## Registration of research studies

NA.

## Guarantor

Mohammad Naser Athamnah.

## Provenance and peer review

Not commissioned, externally peer-reviewed.

## CRediT authorship contribution statement

**Mohammad Naser Athamnah:** Conceptualization, Investigation, Writing - original draft, Writing - review & editing, Visualization, Project administration. **Omar M. Abuelaish:** Supervision, Project administration. **Nimah A. Rabai:** Methodology, Formal analysis, Resources.

## Declaration of Competing Interest

No conflict of interest.

## References

[1] Harris J.R., Lippman M.E., Kent Osborne C., Morrow M. (2012). Diseases of the Breast.

[2] Parker S.J., Harries S.A. (2001). Phyllodes tumours. Postgrad. Med. J..

[3] Reinfuss M., Mituś J., Duda K., Stelmach A., Ryś J., Smolak K. (1996). The treatment and prognosis of patients with phyllodes tumor of the breast: an analysis of 170 cases. Cancer.

[4] Bernstein L., Deapen D., Ross R.K. (1993). The descriptive epidemiology of malignant cystosarcoma phyllodes tumors of the breast. Cancer.

[5] Ben hassouna J., Damak T., Gamoudi A., Chargui R., Khomsi F., Mahjoub S., Slimene M., Ben Dhiab T., Hechiche M., Boussen H., Rahal K. (2006). Phyllodes tumors of the breast: a case series of 106 patients. Am. J. Surg..

[6] White D.S., Irvine T.E. (2013). Rapidly progressive multifocal phyllodes tumour of the breast: a case report and review of the literature. Int. J. Surg. Case Rep..

[7] Mrad K., Driss M., Maalej M., Romdhane K.B. (2000). Bilateral cystosarcoma phyllodes of the breast: a case report of malignant form with contralateral benign form. Ann. Diagn. Pathol..

[8] Chen W.-H., Cheng S.-P., Tzen C.-Y., Yang T.-L., Jeng K.-S., Liu C.-L., Liu T.-P. (2005). Surgical treatment of phyllodes tumors of the breast: retrospective review of 172 cases. J. Surg. Oncol..

[9] Chao X., Chen K., Zeng J., Bi Z., Guo M., Chen Y., Yao Y., Wu W., Liang S., Nie Y. (2019). Adjuvant radiotherapy and chemotherapy for patients with breast phyllodes tumors: a systematic review and meta-analysis. BMC Cancer.

[10] Varghese S.S., Sasidharan B., Manipadam M.T., Paul M.J., Backianathan S. (2017). Radiotherapy in phyllodes tumour. J. Clin. Diagn. Res.: JCDR.

[11] Blanckaert D., Lecourt O., Loeuille G.A., Six J., Laurent J.C. (1988). Phyllodes tumor of the breast in an 11-year-old child. Pediatrie.

[12] Erginel B., Celet Ozden B., Yesil Onder S., Yuksel S., Gun Soysal F., Celik A., Salman T. (2015). Management of a benign phyllodes tumor in a 13-year-old girl with transposition of the nipple areola complex and breast reconstruction. Acta Chir. Belg..

[13] Hassan S., Din N.U., Kayani N. (2016). Malignant phyllodes tumor in an 11-year-old girl with fatal clinical outcome. A case report. Breast Dis..

[14] Kanka K.C., Sawicki J.E., Svahn J.D. (2009). An unusual case of phyllodes tumor presenting as a trans-nipple tumor in a 13-year-old female. Breast J..

[15] Lewitan G., Goldberg C., Serro S.R., Cabaleiro C., Espora S.M. (2010). Phyllodes tumor in an 11 years-old girl: report of a case. Arch. Argent. Pediatr..

[16] Okur M., Erbey F., Bulut G. (2011). Phyllodes tumor in a 16-year-old girl. Pediatr. Hematol. Oncol..

[17] Orea-Estudillo D., Jaimes-López L., Bernal-Cano J. (2008). Phyllodes tumor in a pediatric patient. Case report and literature revision. Cir. Cir..

[18] Sasa M., Morimoto T., Ii K., Tsuzuki H., Kamamura Y., Komaki K., Uyama T., Monden Y. (1995). A malignant phyllodes tumor of the breast in a 6-year old girl. Breast Cancer.

[19] Sorelli P.G., Thomas D., Moore A., Khan M., Hoque H. (2010). Malignant phyllodes tumor in an 11-year-old premenarchal girl. J. Pediatr. Surg..

[20] Abusalem O., AlMasri A. (2011). Phyllodes tumors of the breast. Mater. Socio Med..

[21] Nguyen N.T., Maciolek L.M., Qiu S., Sadruddin S., Nguyen Q.D. (2020). Malignant phyllodes tumor of the breast in a 26-year-old woman. Cureus.

[22] Salm R. (1978). Multifocal histogenesis of a cystosarcoma phyllodes. J. Clin. Pathol..

[23] Hawkins R.E., Schofield J.B., Fisher C., Wiltshaw E., McKinna J.A. (1992). The clinical and histologic criteria that predict metastases from cystosarcoma phyllodes. Cancer.

[24] Mallory M.A., Chikarmane S.A., Raza S., Lester S., Caterson S.A., Golshan M. (2015). Bilateral synchronous benign phyllodes tumors. Am. Surg..

[25] Rajan P.B., Cranor M.L., Rosen P.P. (1998). Cystosarcoma phyllodes in adolescent girls and young women: a study of 45 patients. Am. J. Surg. Pathol..

[26] Khosravi-Shahi P. (2011). Management of non metastatic phyllodes tumors of the breast: review of the literature. Surg. Oncol..

[27] Tremblay-LeMay R., Hogue J.-C., Provencher L., Poirier B., Poirier É., Laberge S., Diorio C., Desbiens C. (2017). How wide should margins be for phyllodes tumors of the breast?. Breast J..

